# Role of Eukaryotic Initiation Factors during Cellular Stress and Cancer Progression

**DOI:** 10.1155/2016/8235121

**Published:** 2016-12-19

**Authors:** Divya Khandige Sharma, Kamiko Bressler, Harshil Patel, Nirujah Balasingam, Nehal Thakor

**Affiliations:** Department of Chemistry and Biochemistry, Alberta RNA Research and Training Institute, University of Lethbridge, Lethbridge, AB, Canada T1K 3M4

## Abstract

Protein synthesis can be segmented into distinct phases comprising mRNA translation initiation, elongation, and termination. Translation initiation is a highly regulated and rate-limiting step of protein synthesis that requires more than 12 eukaryotic initiation factors (eIFs). Extensive evidence shows that the transcriptome and corresponding proteome do not invariably correlate with each other in a variety of contexts. In particular, translation of mRNAs specific to angiogenesis, tumor development, and apoptosis is altered during physiological and pathophysiological stress conditions. In cancer cells, the expression and functions of eIFs are hampered, resulting in the inhibition of global translation and enhancement of translation of subsets of mRNAs by alternative mechanisms. A precise understanding of mechanisms involving eukaryotic initiation factors leading to differential protein expression can help us to design better strategies to diagnose and treat cancer. The high spatial and temporal resolution of translation control can have an immediate effect on the microenvironment of the cell in comparison with changes in transcription. The dysregulation of mRNA translation mechanisms is increasingly being exploited as a target to treat cancer. In this review, we will focus on this context by describing both canonical and noncanonical roles of eIFs, which alter mRNA translation.

## 1. Introduction

Regulation of protein translation is a critical step of the gene expression process, which allows cellular adaptation during stress conditions by rapidly reprograming the proteome output without the requirement for changes in RNA synthesis. In conditions, such as heat shock, hypoxia, endoplasmic reticulum (ER) stress, and apoptosis, an immediate change in protein levels is required, stressing the importance of translational regulation, responsible for rapid adaptation to physiological conditions [1]. Transcriptome analysis is a widely accepted method for analyzing gene expression during stress conditions. However, there is an emerging body of evidence that shows a limited correlation between the transcriptome and the corresponding proteome, suggesting that when it comes to translation, not all transcripts are treated equally. Epidermal growth factor (EGF) treatment of serum-starved HeLa cells resulted in only 4.8% differentially expressed genes (DEGs), where a DEG represents a significant change in both the transcriptome and translatome in the same direction (homodirectionally) [2]. In opposition, the 95.2% of uncoupled DEGs represent a significant change in either the transcriptome or translatome or an inverse relationship between the transcriptome and translatome [2]. Using parallel genome-scale measurements of mRNA and corresponding protein levels and half-lives, mRNAs were found to explain 40% of the variability in protein levels, with translation efficiency being the best predictor of protein levels in mouse fibroblasts [3]. Accordingly, translation control is considered to play a central role in eukaryotic gene expression. As new evidence is being uncovered, scientists have now started to appreciate the critical role of mRNA translation in tumor progression. In a wide range of cancer types, inappropriate translation of oncogenes, tumor suppressors, and eukaryotic translation initiation factors is a critical process in cancer cell proliferation [4–6]. Even during times of stress, when global levels of protein synthesis are reduced, cancer cell development typically involves selective translation of a specific subset of mRNAs. These transcripts encode prosurvival proteins that are translated by alternative (noncanonical) mechanisms [1, 7, 8]. Studies continue to provide new knowledge with regard to the developmental causes and possible novel treatments of various types of cancers [4–7, 9]. Significantly, much of this information can be tied back to the paradigm of translational regulation and its critical contribution to our understanding of cancer etiology.

In this review, we will first discuss the mechanism of canonical translation initiation, followed by noncanonical mechanisms that utilize RNA sequence features including upstream open reading frames (uORFs) and internal ribosome entry site- (IRES-) mediated translation mechanisms, the role of eukaryotic initiation factors in noncanonical translation, and their significance in cancer progression. In addition to uORFs- and IRES-mediated translation regulation, other noncanonical translation mechanisms exist, such as gamma interferon-activated inhibitor of translation (GAIT) complex, 5′ terminal oligopyrimidine (5′ TOP) elements, and AU-rich elements (AREs) [10, 11]. These mechanisms are out of the scope of this review article.

*Canonical Translation Initiation*. Eukaryotic cap-dependent translation initiation includes the recognition and recruitment of mRNA onto the small ribosomal (40S) subunit, followed by ribosomal scanning in a 5′–3′ direction. Subsequently, the 60S large ribosomal subunit is recruited, forming the 80S initiation complex. At this stage, an initiator methionyl-tRNA (met-tRNA*i*) is in the ribosomal peptidyl (P) site at the mRNA start codon [1, 13]. Canonical initiation is a complex process utilizing more than 25 proteins, including a minimum of twelve eukaryotic initiation factors (eIFs) [14]. The rate of initiation varies between different mRNAs and is influenced by accessibility to the methylated guanosine cap structure (m^7^G cap) at the 5′ terminus of the mRNA, the length and secondary structure of the 5′ untranslated region (UTR), the sequence and secondary structure surrounding the start codon, and the poly(A) tail [15, 16].

**Figure 1:**
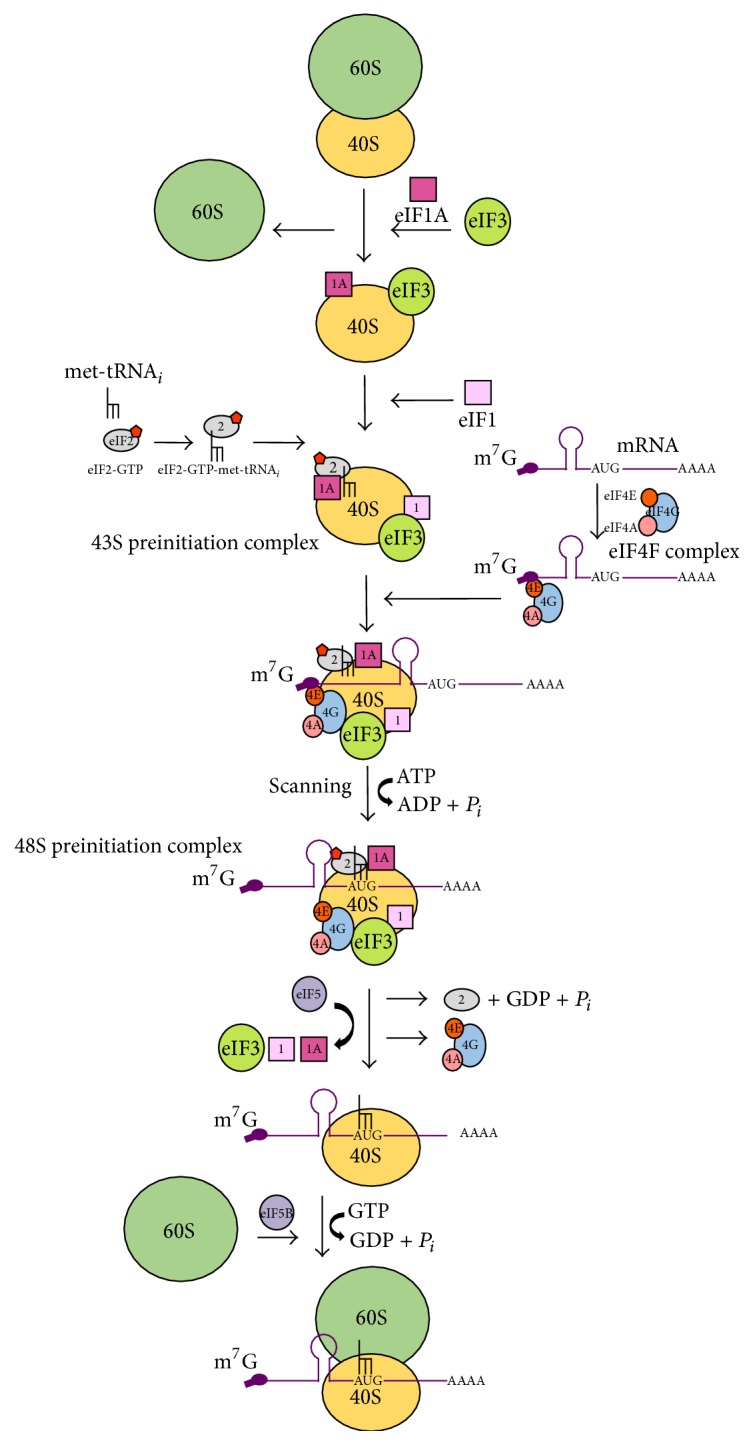
An overview of eukaryotic translation initiation. Most eukaryotic mRNAs contain a 5′m^7^G cap which is bound by eukaryotic initiation factor 4F complex (eIF4E, eIF4G, and eIF4A). The 43S preinitiation ribosome complex which contains ternary complex (Tc) (eIF2-GTP-initiator tRNA) is recruited to the 5′ end of mRNAs via eIF3-eIF4G interaction. With the help of eIF4A (RNA helicase) the preinitiation complex is thought to scan mRNA until the start codon (AUG) is found. Subsequently, the 48S initiation complex is formed and Tc delivers tRNA into the P-site of the ribosome. Then, eIF5 binds to the 48S initiation complex and induces GTPase activity of eIF2*α*. Upon GTP hydrolysis, all protein factors are released from the 40S ribosome subunit. Subsequently, eIF2*α* is recharged with GTP by “GDP to GTP” exchange factor eIF2B. Finally, eIF5B unites the 60S and 40S ribosome subunits to form the 80S initiation complex and translation elongation commences.

Initiation begins with the assembly of eIF4F complex comprising eIF4E, eIF4G, and eIF4A onto the 5′m^7^G cap (Figure 1). eIF4E binds to the m^7^G cap, which then interacts with the multidomain scaffold protein eIF4G and the ATP-dependent RNA helicase eIF4A. The ternary complex (eIF2-GTP-met-tRNA*i*) associated with a 40S ribosomal subunit is then recruited to the 5′ end of the mRNA via a critical link to eIF4G (captured at the cap via eIF4E) mediated by eIF3, forming the 43S preinitiation complex. eIF1 and eIF1A assist in stimulating recruitment of the ternary complex, as well as acting synergistically to promote continuous ribosomal scanning for AUG start codons [17]. This 43S preinitiation complex then scans the 5′ UTR of the mRNA, with the help of eIF4A, until an initiation codon in optimal context is recognized [18]. eIF5 and eIF5B then mediate subsequent hydrolysis of GTP to release the bound initiation factors from the 48S complex, leaving the start codon in the ribosomal P-site with the met-tRNA*i* and allowing 60S ribosomal subunit to bind [1, 19]. The now competent 80S initiation complex then proceeds to translation elongation (Figure 1).

*Noncanonical Translation Initiation*. During stress conditions, such as hypoxia, nutrient deprivation, or endoplasmic reticulum (ER) stress, alternative mechanisms that are mediated by* cis*-acting sequences in specific mRNA subsets, such as uORF and IRES, drive the translation of stress response mRNAs [1, 6–8, 18, 20]. Studies have revealed that a decent portion of human transcripts is known to contain uORFs (upstream of the initiation codon of the coding region) that function as translation or mRNA stability regulators [21]. Recent ribosome profiling data reveals that uORFs can exist in an out-of-frame relative to the main coding sequence [22]. However, an overlap can also occur between uORFs and the coding sequence, in which alternative translation of an upstream in-frame start codon of a gene can possibly produce an extended protein product [23]. The mechanism of uORF-mediated translation functions primarily during eIF2*α* phosphorylation conditions and enhances the expression of proteins involved in cell-cycle regulation and apoptosis. Typical examples of uORF-mediated translation regulation include general control nonderepressible 4 (GCN4), the yeast transcriptional activator, and activating transcription factor 4 (ATF4) in mammals. Normally, translation initiation occurs from the start codons located in the 5′ UTR of mRNA which leads to the translation of small uORFs. Additionally, reinitiation of terminating ribosomes will typically not occur on the downstream cistron; thus, the translation of the main coding sequence is inhibited in these conditions [24]. However, during stress conditions eIF2*α* phosphorylation attenuates translation of uORF sequences and allows the translation of main coding sequence. eIF2*α* phosphorylation and reduced availability of the eIF2-GTP-tRNA*i* ternary complex favor translation reinitiation at the Gcn4p coding region (in yeast), subsequently resulting in activation of numerous genes [25, 26]. Gcn4p activates these target genes by binding to them and functioning as a transcription factor [25]. The complexity of RNA structure in the 5′ UTR also plays a crucial role in uORF-mediated translation. For example, translation of *β*-site APP-cleaving enzyme 1 (BACE1), which is implicated in Alzheimer's disease (AD) progression, is regulated through uORFs. However, high GC content and complexity of the RNA secondary structure are also crucial decisive factors for uORF-mediated translation of BACE1 [27]. Additionally, recent genome-level studies indicate that RNA secondary structure is negatively correlated with uORF translational efficiency as upstream (relative to uORFs) structures restrict or even arrest ribosomal preinitiation complex (PIC), whereas structures downstream of uORFs enhance translation initiation of coding sequences [28].

Translation initiation mediated by IRES is another mechanism that operates during stress conditions. IRESes are RNA sequence elements that were initially discovered in the 5′ leader sequences of poliovirus and encephalomyocarditis virus genomic RNA that lack the 5′ cap structure but nonetheless are efficiently translated in the host cell [18, 29]. The viral IRES elements comprise secondary and tertiary structures that play a role in direct interactions with the translation initiation machinery [20]. Mutations in viral IRESes such as hepatitis C virus (HCV), classical swine fever virus (CSFV), and cricket paralysis virus (CrPV) affect the secondary and tertiary RNA structures and render these IRESes inactive [30]. These viral IRESes are classified based on structural and sequence similarities, as well as their requirement for eIFs and other protein factors for translation initiation [19, 20]. Picornavirus IRES elements are the examples of types I and II IRESes which require eIF4G, eIF4A, and eIF3 to assemble 48S initiation complex [29]. Type III viral IRESes require eIF4G, with HCV IRES being an exception [29]; HCV IRES interacts with eIF3 for recruitment of the 40S ribosomal subunit in close proximity to the start codon, circumventing the requirement for the 5′ cap structure [1, 19]. Although IRES-mediated translation operates independently of many canonical initiation factors, it requires RNA-binding proteins known as IRES* trans*-acting factors (ITAFs).

Many cellular mRNAs are known to comprise IRES elements, but they do not share structural or sequence similarities, unlike their viral counterparts [20, 31]. However, similar to viral IRESes, cellular IRESes participate in multiple interactions with the canonical initiation factors and ITAFs to recruit the ribosome [1, 20]. In fact, despite sequence and structural dissimilarities, cellular IRESes are reported to share critical ITAFs [20]. IRES elements have been identified in mRNAs encoding stress response proteins (pro- and antiapoptotic), such as X-linked inhibitor of apoptosis (XIAP), cellular inhibitor of apoptosis 1 (cIAP1), B cell lymphoma extralarge (Bcl-xL), Bcl-2, Bag-1, apoptotic protease-activating factor 1 (Apaf-1), p53, L-myc, N-myc, c-myc, and death-associated protein 5 (DAP5) [1, 8, 32–34].

## 2. Role of Eukaryotic Initiation Factors in Noncanonical Translation

Translation initiation switches from cap-dependent to IRES-dependent mode during stress conditions such as hypoxia, vascular lesions, serum deprivation, *γ*-irradiation, apoptosis, growth arrest, and angiogenesis [35]. This shift is attributed to eIF2*α* phosphorylation, eIF4E-BP dephosphorylation, and eIF4G cleavage, any of which can inhibit canonical translation initiation [33, 36]. Although the cellular IRES elements are activated under stress conditions, these IRESes differ in their requirement for eIFs. For example, the L-myc IRES requires the eIF4F complex and interaction of both poly(A) tail binding protein (PABP) and eIF3 with eIF4G for translation [20]. On the other hand, partial silencing assays (using the knockdown plasmid, pSilencer31 (si31), and hippuristanol (eIF4A inhibitor) treatment) have demonstrated that C- and N-myc IRESes can function only in presence of the C-terminal domain of eIF4GI, eIF4A, and eIF3; This IRES does not require full-length eIF4GI or PABP [34].

ER stress caused by the accumulation of unfolded proteins triggers the unfolded protein response (UPR) which in turn modulates both transcription and translation of key regulators (e.g., ATF4) of the cellular stress response [37]. A recent study has shown that an alternatively spliced variant of the previously discussed human ATF4 transcript (variant V1) is translated by an IRES-mediated mechanism [37]. This variant also has a unique 5′ leader sequence and is found to be less abundant than other variants in numerous human tissues. Interestingly, these variants were shown to be translated by different mechanisms by using luciferase reporters and modifying 5′ leader sequences with stem-loop insertions. Chan et al. found high GC content in the long and highly structured 5′ leader region of V1 in comparison to V2, suggesting a possibility of IRES-mediated translation. Testing truncated versions with bicistronic reporter assay, Chan et al. found structural elements that likely interact with* trans*-acting cellular factors. Additional tests involving inhibition of critical canonical eIFs (e.g., eIF4G1) and eIF2*α* phosphorylation indeed confirmed the IRES-mediated translation of V1 during UPR [37].

**Figure 2:**
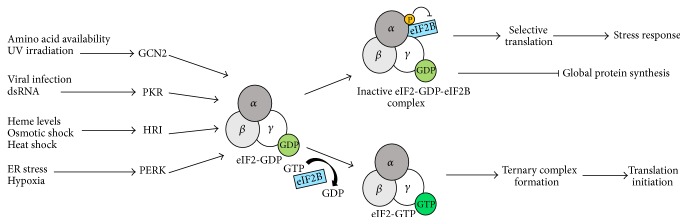
Global translation inhibition by eIF2*α* phosphorylation. Several stress stimuli activate distinct protein kinases, which in turn phosphorylate eIF2*α*. The phosphorylation of eIF2*α* enhances the affinity of eIF2-complex (*α*, *β*, and *γ* subunits) for eIF2B. This renders eIF2-complex inactive for the initiator tRNA delivery to the ribosome. However, a subset of mRNAs harboring* cis*-elements such as internal ribosome entry site (IRES) or upstream open reading frames (uORFs) are preferentially translated during eIF2*α* phosphorylation conditions. These mechanisms allow production of stress-related proteins. GCN2: general control nonderepressible-2; PKR: protein kinase R; HRI: heme-regulated inhibitor kinase; PERK: PKR-like endoplasmic reticulum kinase.

**Figure 3:**
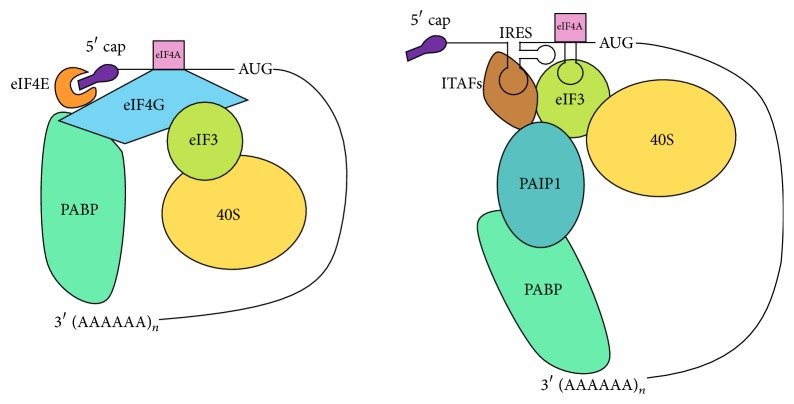
Comparison between cap-dependent and IRES-dependent translation initiation of cellular mRNAs. In cap-dependent translation, initiation complex is formed on the 5′ end of mRNA with help of several eukaryotic initiation factors (eIFs). eIF4F-complex (eIF4E, eIF4G, and eIF4A) is recruited to the 5′m^7^G cap. The key interaction between eIF3 and eIF4G recruits the ribosome to the 5′ cap. In IRES-mediated translation, initiation complex is formed in the vicinity of the start codon with help of ITAFs and eIFs. For example, XIAP IRES interacts with eIF3 and recruits the ribosome via eIF3-PAIP1-PABP link [12]. IRES: internal ribosome entry site; PABP: poly(A) tail binding protein; PAIP1: PABP interacting protein 1; 40S: 40S ribosomal subunit; AUG: start codon.

During amino acid starvation, viral infection, or endoplasmic reticulum stress, several kinases are activated that induce eIF2*α* phosphorylation (Figure 2). This phosphorylation, in turn, decreases eIF2-GTP-tRNA*i* ternary complex activity, resulting in suppression of cap-dependent translation. However, several viral and cellular mRNAs are insensitive to this mode of translation inhibition [20]. This suggests that mRNAs can employ alternative factors or mechanisms to recruit the eIF2-GTP-tRNA*i* ternary complex. Examples of cellular IRESes unaffected by eIF2*α* phosphorylation are XIAP, c-myc, cationic amino acid transporter-1 (cat-1), and N-myc [38]. Many viral IRESes also bypass translation inhibition exerted by eIF2*α* phosphorylation. The exact mechanism by which the consequences of eIF2*α* phosphorylation are avoided by the HCV IRES is not known. However, some IRESes (CSFV and HCV) employ eIF5B, an orthologue of prokaryotic IF2, during conditions of increased eIF2*α* phosphorylation [30]. The eIF5B-eIF3 mediated mechanism involves eIF3 stimulating tRNA*i* binding to the 40S subunit (in the IRES/40S complex) in an eIF5B-dependent manner, which allows for the formation of the 48S initiation complex and, subsequently, the translation-competent 80S ribosome [30]. Like IF2, eIF5B binds and delivers initiator tRNA during translation initiation on these IRESes [8, 30]. We have recently found that the XIAP IRES uses a similar mechanism during eIF2*α* phosphorylation conditions. This finding suggests that eIF5B-dependent activation of IRES-mediated XIAP mRNA translation would act as a critical prosurvival switch in cells under stress [8]. Moreover, a recent publication from our lab suggests that the XIAP IRES does not require eIF4G, eIF4E, and eIF4A for initiation complex formation. The inhibition of eIF4A activity by hippuristanol or pateamine A treatment did not affect the ability of XIAP IRES to form initiation complexes, suggesting eIF4A is not required for IRES-mediated translation of XIAP [8]. Additionally, eIF3 and PABP bind synergistically with in vitro-transcribed, uncapped, and poly(A)-tailed XIAP IRES RNA and recruit ribosomes near the start codon [12] (Figure 3). The XIAP IRES adopts a conformation that is critical for ribosome recruitment. Although cellular IRESes do not share structural similarities in studies conducted thus far, the secondary structure is indeed important as we have found it to be required for efficient recruitment of eIF3 and the ribosome [12].

A recent study suggests that eIF3 subunit d plays a key role in alternative mechanisms of translation initiation by a noncanonical mechanism of cap recognition. Specifically, the study classifies this method as a novel, eIF4E-independent mechanism of translation initiation [39]. Lee et al. found that the eIF3d subunit alone provided RNase protection to the mRNA of the early response transcription factor c-Jun by directly interacting with a mature, methylated 5′ cap structure. The specificity of eIF3d binding to only a subset of capped mRNAs is suggested to occur through an “RNA gate” domain that regulates this novel cap-binding function of eIF3d. In the larger context of eIF3 specialized translation pathways, eIF3d seems to play a critical role as a cap-binding protein that helps cells regulate protein synthesis even during times when the eIF3F complex (required for canonical translation) is inactivated or inhibited [39].

Mitochondria play an important role in the intrinsic pathway of apoptosis, which is regulated by the Bcl-2 family of proteins. For example, Bax and Bac proteins activate mitochondria-dependent apoptosis, whereas Bcl-xL and Bcl-2 proteins inhibit apoptosis [40]. During apoptosis, Bcl-xL expression is controlled by sulphated glycoprotein 2 (SGP-2). The phosphorylation levels of eIF4E and 4E-BP1 are influenced by SGP-2 which, in turn, affects the Bcl-xL IRES-dependent translation by regulating the stability of eIF4F complex [41]. Overexpression of some protooncogenic proteins (Bcl-xL and c-myc), as implicated in numerous cancer types, could be a result of elevated levels of eIF4F [41].

DAP5 (p97), eIF4G1, and eIF4G2 comprise the eIF4G family and function in the formation of the translation initiation complex. DAP5 domains share homology with the central and C-terminal region of eIF4G that interacts with eIF4A, eIF3, Mnk1, and eIF2*β* but not eIF4E [42]. The interaction of DAP5 with eIF2*β* and eIF4AI facilitates the IRES-mediated translation of various cellular mRNAs, including those encoding pro- and antiapoptotic proteins, such as c-Myc, Bcl2, Apaf1, XIAP c-IAP1, and DAP5 itself [42, 43]. Moreover, the cleavage of DAP5 by caspase generates a smaller fragment, p86, which also facilitates the IRES-mediated translation of various cellular mRNAs [44]. The cleavage of eIF4GI during apoptosis yields three major cleavage products: N-FAG (N-terminal Fragment of Apoptotic Cleavage of eIF4G), M-FAG (Middle-FAG), and C-FAG (C-terminal FAG); the cleavage causes disassembly of the eIF4F complex as the fragments no longer retain the ability to bind eIF4E, eIF4A, and eIF3. Surprisingly, eIF4G M-FAG alone supports the IRES-mediated translation of mRNAs, including p97/DAP5, XIAP, and c-IAP1 [32].

During angiogenesis, IRES expression of the potent angiogenic factor, vascular endothelial growth factor (VEGF), is regulated by its interaction with eIF4E and eIF4G. The direct interaction of eIF4G1 with VEGF IRES enhances its translation during breast cancer progression [45]. Knockdown of eIF4GI using RNA interference in a chick chorioallantoic membrane system resulted in decreased VEGF protein levels and a reduction in angiogenesis [45]. This reduction was specific to IRES-mediated translation, as silencing of eIF4GI had no drastic effect on global rates of protein synthesis in normoxic conditions. The depletion of eIF4GI attenuated global mRNA translation rates, suggesting a bigger requirement of eIF4GI during hypoxic conditions [45]. The C-terminus of eIF4GI stimulates cap-independent translation initiation at the 5′ UTR of c-myc and VEGF. Under hypoxic conditions, VEGF, FGF-2, Bcl-2, and hypoxia-inducible factor 1*α* (HIF1*α*) are all overexpressed due to upregulation of IRES-mediated translation [46, 47]. This selective translation is mediated by the overexpression of eIF4E-BPs and eIF4G and is particularly advantageous for cancer cells as VEGF, FGF-2, HIF1*α*, and Bcl-2 are all significant factors in promoting tumor growth and survival [45, 48].

## 3. IRES* trans*-Acting Factors Regulating IRES-Mediated Translation

The ITAFs are protein factors that interact specifically with the IRES based on the sequence and structure of the mRNA and modulate IRES-mediated translation. ITAFs can act as a molecular chaperone or modify the structure of RNA to facilitate direct recruitment of the eukaryotic initiation factors and the ribosome to form 48S initiation complex. Some of the well-characterized ITAFs are human antigen R (HuR), La autoantigen, programed cell death 4 (PDCD4), polypyrimidine tract binding (PTB) protein, heterogeneous nuclear ribonucleoprotein A1 (hnRNPA1), hnRNAC1/C2 upstream of NRAS (UnR), nuclear factor 45 (NF45), insulin-like growth factor 2-binding protein 1 (IGF2BP1), Y-box protein 1 (YB1), and poly(C) binding protein (PCBP) [20, 49, 50]. The levels, activity, and localization of these ITAFs are regulated by various signaling pathways, which in turn regulate the IRES-mediated translation. Hence many of these ITAFs are implicated in tumor cell survival and cancer progression.

There are two classes of ITAFs: one that facilitates while the other represses IRES-dependent translation. For example, NF45 promotes while hnRNPA1 inhibits the IRES-mediated translation of XIAP [51, 52]. HuR directly interacts with the XIAP and Bcl-xL IRES and modulates their translation. HuR interacts with the XIAP IRES through the RNA recognition motifs, RRM1 and RRM2, to stimulate protein synthesis. On the contrary, interaction with the Bcl-xL IRES decreases translation and enhances the membrane integrity of mitochondrial promoting cell survival [53, 54]. La autoantigen as part of RNP complex enhances XIAP IRES-mediated translation as demonstrated by in vitro and in vivo assays [55].

PDCD4 had been considered the general translation inhibitor which sequesters eIF4A and inhibits its helicase activity [56]. However, it is shown that the direct interaction of PDCD4 with the XIAP IRES is required to inhibit the IRES-mediated translation of XIAP. PDCD4 in the absence of activated S6K2 directly binds to the XIAP and Bcl-xL IRES and blocks 48S preinitiation complex formation [57]. The activation of S6K2 by the fibroblast growth factor 2 (FGF2) results in phosphorylation of PDCD4 and subsequent removal by the proteasomal pathway, which in turn upregulates IRES-mediated translation of XIAP and Bcl-xL [57].

PTB protein forms ribonucleoprotein complexes with PSF, hnRNPL, and other proteins to regulate the gene expression, stability, and localization of mRNA during apoptosis. The complex formed is constantly remodeled depending on the type of apoptotic stimulant. When TRAIL activates apoptosis, PTB protein forms complex with PSF, YBX1, NONO/p54nrb, hnRNPA2/B1, hnRNPC1/C2, and DDX3X and regulate the IRES activity of mRNAs involved in apoptosis. The interactions could occur in the nucleus prior to splicing and in the cytoplasm aiding recruitment of the ribosome. Cytoplasmic translocation of PTB protein leads to increase in TRAF1, p53, and p47 mRNA expression. Specific mutation of cytosine to thymidine observed in domain 2 of c-myc IRES derived from multiple myeloma cell lines demonstrated enhanced interactions with PTB protein and Y-box binding protein 1. This increase in protein binding is correlated with an elevated IRES activity of c-myc mRNA in multiple myeloma. In human melanoma, single nucleotide polymorphism at the PTB protein binding site present on p53 5′ UTR decreased IRES activity, emphasizing the importance of PTB protein as an ITAF [58–62].

Overexpression of hnRNPC activates XIAP IRES activity with no effect on cap-dependent translation [63]. Also, hnRNPC1/C2 interaction with the p53 IRES is critical for mRNA expression and thus affects transcription of proapoptotic mRNA [64, 65]. Moreover, hnRNPC interacts with the heptameric uridine sequence in the c-myc IRES and enhances c-myc expression only during G2/M phase of cell cycle [66]. hnRNPA1, which is part of hnRNP family of proteins, regulates expression of Bcl-xL and XIAP mRNA. Phosphorylation at the RRM1 domain of hnRNPA1 by S6K2 selectively promotes association of Bcl-xL or XIAP with hnRNPA1 and exports the RNA-protein complex into the cytoplasm. hnRNPA1 interaction with the mRNA suppresses its IRES activity. The suppression is relieved by sumoylation of the RRM2 domain of hnRNPA1, resulting in the decreased affinity of hnRNPA1 to protein and translocation into the nucleus [67].

Unr acts as either a positive or a negative regulator of apoptosis depending on the cell type. Deleting unr in the embryonic stem cells resulted in decreased expression of p53, Gadd45g, and caspase-3, impairing apoptosis signaling pathway in response to gamma irradiation, whereas when unr expression was partially silenced, induction of apoptosis was observed [68]. Unr interaction with the Apaf1 IRES opens the stem-loop structure of IRES enabling PTB protein binding. PTB proten and unr act synergistically as chaperones to create a single-stranded region for the recruitment of 48S complex [69]. PCBP like unr unwinds Bag-I IRES and facilitates landing of the ribosome. Besides the mentioned positive regulator, ITAFs such as hnRNPC1 and nucleolin can negatively regulate p53 expression [49]. In conclusion, the levels and localization of ITAFs in the cell are critical for regulation of gene expression. During stress conditions, proteins are modified mostly by phosphorylation reactions that trigger nuclear to cytoplasmic localization and modulate IRES-mediated translation.

## 4. Role of Eukaryotic Initiation Factors in Canonical Translation

### 4.1. eIF2

eIF2 is a heterotrimeric protein, composed of *α*, *β*, and *γ* subunits [70]. eIF2 is a required element of the ternary complex that delivers met-tRNA*i* to the 40S ribosomal P-site in translation initiation [71]. eIF2 exists in a GDP- or GTP-bound configuration, which has a critical role in translational control during stress [71]. During translation initiation, eIF2 bound GTP hydrolysis is induced by eIF5, releasing eIF2-GDP in the inactive form. eIF2B catalyzes the exchange of GDP to GTP which is necessary for reformation of the ternary complex [70]. Under stress conditions such as an excess of unfolded proteins in the endoplasmic reticulum, or amino acid starvation, *α* subunit of eIF2 is phosphorylated at serine 51 by one of four members of the eIF2*α* kinase family [72, 73]. Phosphorylation of eIF2*α* sequesters eIF2B, locking eIF2 and eIF2B in an inactive complex [71]. Inhibition of active eIF2-GTP regeneration results in decreased ternary complex thus inhibiting overall translation (Figure 2). Furthermore, eIF2*α* phosphorylation has a role in suppression of tumorigenesis, demonstrated by the ability of protein kinase RNA-activated (PKR) to promote malignant transformation of NIH 3T3 cells [70, 74]. The transformation mechanism inhibits eIF2*α* phosphorylation by decreasing the activity of upstream target PKR, potentially through the formation of inactive PKR heterodimers [70, 74]. Decreased levels of phosphorylated eIF2*α* were found in osteosarcoma tumors, while increased PKR levels and associated phosphorylated eIF2*α* levels were correlated with tumor cell differentiation [74, 75]. Expression levels of phosphorylated eIF2*α* serve as a marker for determining the prognosis of non-small lung cancer (NSCLC) patients [76].

ER stress is closely associated with solid tumor progression, having implications in cancer proliferation and apoptosis. Downstream mediators and targets of ER stress include activating transcription factor 6, inositol-requiring enzyme 1 (IRE1), and protein kinase RNA-like ER kinase (PERK) which is an upstream activator of eIF2*α* phosphorylation [77, 78]. ER stress is induced in chronic myeloid leukemia (CML), which activates PERK and eIF2*α* phosphorylation [79]. Phosphorylation of eIF2*α* supports CML progression with a prosurvival role, shown in the inhibition of PERK, which prevents eIF2*α* phosphorylation [79]. This, in turn, allows sensitization of CML cells to imatinib and decreases their proliferative abilities [79]. Insulin-like growth factor binding protein-5 (IGFBP-5) and protein family member IGFBP-3 upregulate expression of growth arrest and DNA damage-inducible protein 34 (GADD34), which assembles an eIF2*α* dephosphorylation complex, enabling regeneration of active eIF2, critical to ternary complex formation [80]. GADD34's dephosphorylation activity is required to recommence protein synthesis after periods of global translation inhibition. However, translation of uORF-containing, prooncogenic protein ATF4 is upregulated by eIF2*α* phosphorylation, promoting osteogenesis and osteoblast differentiation [80]. In ovarian cancer cells, autophagy and activation of the PERK/eIF2*α* pathway attenuate and protect cancer cells from metformin-induced apoptosis [81]. In contrast, protein Obg-like ATPase 1 (OLA1) inhibits protein synthesis and promotes integrated stress response without utilizing eIF2*α* phosphorylation [82]. OLA1 is a GTPase that binds to eIF2, preventing ternary complex formation [82]. In vivo, OLA1-knockdown inhibits the mainly prosurvival integrated stress response (ISR) pathway in cancer cells, which is responsible for restoring cellular homeostasis in response to physiological changes as well as intrinsic stresses such as ER stress [82, 83]. Inhibition of the ISR pathway results in attenuated CCAAT-enhancer-binding protein homologous protein (CHOP) levels and promotion of tumor growth and metastasis through cell proliferation [82]. CHOP expression is triggered by unfolded protein accumulation in the endoplasmic reticulum (ER) [84]. CHOP induces apoptosis during prolonged stress or stress response malfunction, through the formation of heterodimers with other C/EBP family members [84]. Thereby, attenuation of CHOP results in inhibition of apoptosis in stress conditions.

Besides phosphorylation of eIF2*α*, increased levels of eIF2*α* expression are detected in tumor samples in bronchioloalveolar carcinomas of the lung, Hodgkin's lymphoma, gastrointestinal carcinomas, malignant melanoma, and melanocytic neoplasms [70, 85]. eIF2*α* levels were highly expressed, along with eIF4E in the germinal centers of reactive follicles when examined in several types of non-Hodgkin's lymphoma [86]. Comparably, eIF2*α* and eIF4E were present in the nuclei and cytoplasm of brain tumor cells, with a higher concentration of eIF2*α* in the nuclei of gastrointestinal cancer tumor cells [87, 88]. Differential expressions of eIF2*α* may relate to abnormal protein synthesis, furthering its role in tumorigenesis [85]. As the main effector of both global translation and translation of specific subsets of mRNAs, eIF2 continually shows potential for cancer therapeutics and treatments.

### 4.2. eIF3

Eukaryotic initiation factor 3 (eIF3) is a 13-subunit complex of 800 kilodaltons, required for translation initiation through interactions with the 40S ribosomal subunit, mRNA, and other eIFs necessary for the formation of competent translation initiation complexes [19, 114]. eIF3, along with eIF1A and plausibly eIF5, associates with the ternary complex and the 40S ribosomal subunit to form the 43S preinitiation complex. eIF3 enhances the stability of 43S preinitiation complex through eIF3-eIF4G interaction [1, 19, 89]. Primarily, eIF3j regulates eIF3 interaction with the mRNA-binding cleft on the 40S subunit by inhibiting mRNA entry and confirming met-tRNA*i* is present in the P-site [115–117]. Interestingly, beyond the protein synthesis related functions of the eIF3 complex, dysregulation of eIF3 subunits has been implicated in several types of cancers [70, 90]. Variations in the levels and activity of eIF3 subunits are a result of upstream signaling molecules such as protein kinases, involving phosphorylation of eIF3 subunits. For example, the mammalian target of rapamycin complex 1 (mTORC1), a key protein kinase involved in the regulation of protein synthesis, facilitates interaction between PAIP1 and eIF3 [111]. The eIF3g subunit directly interacts with PAIP1 in an RNA-independent manner, which enhances PAIP1-mediated translation stimulation in vivo [111, 118]. Stimulation of mTORC1 (e.g., by amino acids) phosphorylates S6K, which ultimately stimulates interaction between eIF3 and PAIP1. This PAIP1-eIF3 interaction is also proposed to stabilize the conformation of circularized mRNA by stimulating the eIF4G-PABP interaction [111, 118]. Moreover, mTOR inhibition by rapamycin and PP242 (inhibitor of mTORC1 and mTORC2) significantly decreased S6K1 phosphorylation and subsequently diminished this PAIP1-eIF3 interaction [111, 118]. Evidence for the direct association of mTOR and S6K1 with eIF3 points to probable effects on translation. Specifically, studies have shown that the eIF3 complex, as found on the translation preinitiation complex (eIF3-PIC), functions as a scaffolding structure which is associated with mTOR by mitogen/hormone stimulation, whereas S6K1 dissociates from this complex upon similar stimulation [94]. Immunoprecipitation experiments in the immortalized human embryonic kidney cells (HEK293E/T) have shown that S6K1 associates with eIF3b, eIF3c, eIF3e, and eIF3f and mTOR coimmunoprecipitates with eIF3c [94, 111]. HEK293T cells are derived from the original HEK293E cell lineage and have been modified to allow for transient transfection of vectors containing the SV40 origin of replication [119]. Interestingly, in nutrient-deprived conditions, the S6K1-eIF3 association is observed, whereas the addition of amino acids diminishes this direct interaction [94]. Notably, with insulin treatment, S6K1 dissociates from the eIF3-PIC, whereas mTOR is associated with this complex. This insulin stimulation results in an increase in cap-dependent translation, suggesting the mTOR-eIF3-PIC association and the subsequent series of phosphorylation events are critical for efficient protein synthesis [94].

In addition to the critical function of the eIF3 complex in translation initiation, many eIF3 subunits have been shown to be involved in a diverse set of cellular processes including apoptosis, oncogenesis, and cellular growth and proliferation [89, 100, 109, 114, 120–122]. Upregulation of eIF3a, eIF3b, eIF3c, eIF3d, eIF3e, eIF3h, and eIF3i along with reduced levels of eIF3e and eIF3f has been observed in several cancers (Table 1). Most notably, increased levels of the largest eIF3 subunit, eIF3a, have been found in breast, cervix, esophagus, lung, and stomach cancers [89, 90]. The mechanism by which upregulation of eIF3a promotes the malignant phenotype in lung cancer (H1299) and breast cancer (MCF7) cells involves enhancing DNA synthesis for maintaining cell proliferation. During the S phase of the cell-cycle, eIF3a upregulates the translational expression of ribonucleotide reductase M2, which in turn maintains high levels of DNA synthesis [89, 91]. When eIF3a is downregulated by using antisense eIF3a cDNA, ribonucleotide reductase M2 levels (and, subsequently, DNA synthesis) are significantly reduced. Furthermore, independently downregulating ribonucleotide reductase M2 expression levels has shown to reduce the extent of malignancy in human cancer cells [91]. These findings delineate the mechanism of how high expression levels of eIF3a maintain cell proliferation in cancer cells. Additionally, eIF3a is also known to enhance phagocytosis during apoptosis by facilitating the association between apoptotic cells and macrophages [122].

Overexpression of eIF3b has been observed in breast, bladder, and prostate cancers; however, the specific mechanism through which upregulated eIF3b promotes the cancer state is still unclear [90]. Increased eIF3c transcript levels have been found in human testicular seminomas [100] as well as increased eIF3c gene expression in colon cancer cells [98]. Interestingly, eIF3c also directly binds to the neurofibromatosis 2 (NF2) tumor suppressor protein, schwannomin, in STS26T schwannoma cells [99]. Schwannomin is thought to employ its tumor suppressive functions by binding eIF3c and inhibiting eIF3c-mediated cell proliferation, possibly due to the role of eIF3c during protein translation initiation. Additionally, in meningiomas, which have significantly reduced levels of schwannomin, eIF3c is upregulated, suggesting a role of eIF3c in tumor growth and proliferation [99]. Furthermore, overexpression of eIF3c or eIF3h resulted in the enhanced translation of cell proliferation mRNAs encoding growth-regulating cyclin D1, c-Myc, fibroblast growth factor 2, and ornithine decarboxylase (ODC) [100]. ODC serves as a marker for cell proliferation and functions as an oncoprotein [123]. This enhancement of translation rates for these proteins and subsequent production of malignant phenotypes may not be a direct consequence of the overexpression of a single eIF3 subunit since enhanced levels of other eIF3 subunits (a, b, c, f, h, and j) were also noted. Thus, the overexpression of eIF3a, eIF3b, or eIF3c subunits stimulates the expression of other eIF3 subunits that further supports the translational components necessary for faster cancer cell growth [100].

Like other eIF3 subunits, eIF3d is also involved in protein synthesis and has been shown to be upregulated in gastric cancer and mesothelioma [70, 104]. Studies using lentivirus-mediated RNA interference to knockdown eIF3d in the colon (HCT116) and non-small cell lung cancer cells (NSCLC—A549 and 95D) showed significantly reduced cell proliferation (induced apoptosis) and inhibited colony formation due to induced cell-cycle arrest in the G2/M phases. Notably, in HCT116 cells, eIF3d knockdown resulted in phosphorylation of AMPK*α*, Bad, PRAS40, SAPK (stress-activated protein kinase)/JNK, GSK3*β*, and PARP [poly(ADP-ribose) polymerase] cleavage. Phosphorylation of Bad, a proapoptotic protein, induces apoptosis while phosphorylation of GSK3*β* promotes reovirus-induced apoptosis. Furthermore, PARP cleavage is typically used as a signal for apoptosis induction [104]. eIF3d knockdown in NSCLC cells resulted in decreased phosphorylation and thus inhibition of AKT, HSP27, and SAPK/JNK (involved in cellular growth and cancer progression pathways). This data supports the crucial role of eIF3d in cell proliferation and cancer growth [104, 105].

In breast cancer cells, reduction of eIF3e expression by RNAi induces EMT (epithelial-to-mesenchymal transition), suggesting a role of eIF3e in breast cancer metastasis. This study and others [108, 124] suggest that eIF3e normally functions as a tumor suppressor since the reduction of its expression results in enhanced mRNA stability and expression of the transcription factors and EMT regulators, Snail1 and Zeb2. This suggests that the loss of eIF3e directly results in cancer progression and metastasis as EMT is induced in breast cancer cells [124]. Conversely, another report has shown that eIF3e functions as an oncogene [125]. In this study, the knockdown of eIF3e using RNAi in U2OS and MDA-MB-231 resulted in a reduction in protein levels of Bcl-xL (antiapoptotic protein) and urokinase-type plasminogen activator (PLAU) but an increase in MAD2L1 (mitotic checkpoint component). Overabundant Bcl-xL protein levels are associated with chemoresistance in cancers while PLAU functions in promoting metastasis in tumors. Interestingly, Bcl-xL mRNA associates directly with the eIF3 complex in an eIF3e-dependent manner as determined by RNA IP. Following eIF3e knockdown, the specific changes in protein levels of the mentioned eIF3e targets, without any changes in global protein synthesis, suggest that eIF3e specifically regulates translation of mRNAs involved in tumorigenesis [125]. Furthermore, eIF3e gene silencing using siRNA in glioblastoma results in cell-cycle arrest in the G1 phase, decreases cell proliferation, and induces both caspase-dependent and caspase-independent apoptosis [126].

Furthermore, a recent study suggests the role of eIF3d-eIF3e module within the eIF3 complex that regulates the translation of specific mRNAs involved in maintaining metabolic pathways that are likely disrupted in cancer cells [127]. Critical components of the mitochondrial electron transport chain were downregulated in both yeast and mammalian cells (nontumorigenic: MCF-10A and nontumorigenic: MCF7) that were eIF3e-depleted using siRNA, whereas glucose metabolism and amino acid biosynthesis processes were upregulated. Their findings suggest that depletion of eIF3e triggers a metabolic switch that increases dependence on glycolysis, as respiratory deficiencies alongside increased sensitivity to oxidative stress are also observed when eIF3d is depleted in addition to eIF3e knockdown. Essentially, this data suggests that the novel function of eIF3d-eIF3e in maintaining mitochondrial respiration components and serving to adjust metabolic pathways may help us better understand how the cancer-promoting properties of the eIF3 complex emerge [127].

Unlike eIF3e, eIF3f is consistently shown to function as a tumor suppressor in pancreatic cancer [109]. Endogenous levels of both eIF3f mRNA and protein levels are reduced in pancreatic cancer cells [128], whereas eIF3f-overexpressing NIH3T3 cells have shown reduced cell proliferation and induced apoptosis [100]. Likewise, eIF3f knockdown in normal human pancreatic epithelial cells has shown an increase in cell proliferation and increased resistance to apoptosis [109]. By utilizing a bicistronic luciferase report system, it was shown that eIF3f normally inhibits both cap-dependent and cap-independent (i.e., IRES) mechanisms of translation initiation. Furthermore, one mechanism of translation inhibition likely involves eIF3f-mediated rRNA degradation by a direct eIF3f-hnRNP K (RNA-binding protein) interaction [109]. Specifically, when eIF3f is present, it binds to hnRNP K, preventing it from binding to rRNA, which subsequently is degraded, and the translation is reduced. In cancer cells, the loss of eIF3f results in an increased binding of hnRNP K to rRNA, reducing rRNA degradation, and possibly favoring oncogenesis through increased translation [109]. These findings suggest that eIF3f functions as a negative regulator of cell growth due to the naturally reduced levels of eIF3f contributing to cancer development and overexpression resulting in apoptosis [128].

Despite the vast quantity of correlative evidences between eIF3 subunit expression levels and observed cancer phenotypes, it has been difficult to establish a clear mechanism of how cancer progression is directly affected by eIF3 expression. Understanding the interactions of the eIF3 subunits with antiapoptotic proteins, cellular growth and proliferation proteins, and oncogenic proteins may provide a better understanding of how eIF3 subunits contribute to supporting or inhibiting the oncogenic state. A functional mechanism will most likely involve aspects of translation initiation, preinitiation complex formation, and other processes, such as the recruitment of the preinitiation complex to the cellular mRNA and ribosome scanning, all of which are mediated to some degree by a subset of eIF3 subunits. Additionally, studies depicting eIF3 as both an activator (for the protooncogene c-Jun) and repressor (for the negative regulator of cell proliferation BTG1) of cap-dependent translation, mediated by binding to specific RNA structural elements [114], illustrate the diversity of functions carried out by the eIF3 complex through interactions with proteins and nucleic acids. Future studies will need to focus on determining the signaling pathways that are involved in regulation of eIF3 and the consequences of eIF3-directed therapeutics for human cancers. This information will assist in the development of novel therapeutics that target eIF3 subunits in order to treat cancers and possibly other human diseases.

### 4.3. eIF4F Complex

eIF4E interacts with the mRNA cap structure and eIF4G for efficient translation. The interaction with eIF4G is inhibited by binding of eIF4E-binding protein (4E-BP) to eIF4E [129]. eIF4G and 4E-BP are known to compete for a common binding site on eIF4E. The eIF4E and 4E-BP interaction is highly regulated via a phosphorylation reaction and acts as a primary target for hindering translation initiation during stress conditions [130]. Activation of the mTOR pathway phosphorylates 4E-BP and enhances cap-binding efficiency of eIF4E. In contrast, inhibition of the mTOR pathway by amino acid starvation dephosphorylates 4E-BP, resulting in increased association between 4E-BP and eIF4E, thus repressing cap-dependent translation. Overexpression of 4E-BP leads to a decrease in the mRNA and protein levels of cyclin D1 and an increase in p27, a cell-cycle regulatory protein that promotes cell-cycle arrest in breast cancer cell lines. Additionally, eIF4E has been demonstrated to have distinct nuclear and cytoplasmic roles. It localizes to the nuclear bodies by interacting with the eIF4E-transporter protein (4E-T). In the nuclear bodies, it remains associated with promyelocytic leukemia proteins that share a common binding site with 4E-T and 4E-BP. Thus, an increase in 4E-BP may affect the mRNA transport function of eIF4E from the nucleus to the cytoplasm, resulting in differential expression of cyclin D1. Also, dysregulation of 4E-BP phosphorylation is correlated with poor prognosis in lung cancer, breast cancer, melanoma, cervical carcinoma, and astrocytoma [131, 132]. eIF4E is phosphorylated at Ser209 by p38 mitogen-activated protein kinase (MAPK). p38 MAPK phosphorylates Mnk, a serine/threonine kinase, and enables Mnk interaction with eIF4G and phosphorylation of eIF4E [133]. Induction with transforming growth factor *β* (TGF*β*) increases eIF4E phosphorylation alongside mesenchymal markers such as N-cadherin, fibronectin, and vimentin, as a result of the noncanonical signaling pathway. As a downstream target of p38, Mnk1 is activated. Mnk1 phosphorylates eIF4E to specifically translate mRNAs transcribed by the canonical SMAD pathway. This eventually leads to the upregulation of SNAIL and matrix metalloproteinase 3, promoting cell invasion and metastasis [134]. Phosphorylation of eIF4E is not an absolute requirement for translation but is observed to increase the rate of translation initiation [129]. Genome-wide studies of translating mRNA have indicated that eIF4E phosphorylation is necessary for synthesizing proteins essential for tumorigenesis, and the levels of eIF4E are critical for antiapoptotic protein expression [135–137].

In nasopharyngeal carcinoma, the latent membrane protein 1 enhances transcription of many oncogenes such as VEGF, c-Myc, and matrix metalloproteinases (MMP). eIF4E promoter activity is enhanced by c-Myc and, as a feedback mechanism, eIF4E increases translation of these oncogenes [138]. Further, overexpression of eIF4E leads to increased expression of a subset of proteins, influencing angiogenesis and tumor progression (VEGF and fibroblast growth factor-2 (FGF-2)), growth stimulation (platelet-derived growth factor), prosurvival (Bcl-2 and Bcl-xL), cell-cycle progression (c-myc, cyclin D1, and ornithine decarboxylase), epithelial-to-mesenchymal transition (SNAIL and MMP), and invasion (integrin *β*1) [134, 137, 139–142]. Besides the role of eIF4E as a cap-binding protein, it is reported to stimulate the helicase activity of eIF4A and aid translation of mRNAs comprising long, structured 5′ UTRs [143].

A subset of transcripts harbors a 12-nucleotide motif (CGG)_4_ called the G-quadruplex in their 5′ UTRs. Some examples of mRNAs including such structures are VEGF, Bcl-2, and NRAS. These mRNAs are sensitive to silvestrol, a drug that inhibits the helicase activity of eIF4A, thus indicating the role of eIF4A, discrete from mTOR-dependent translation regulation [144–146]. Ribosome footprinting and polysome profiling experiments have detected more than 250 genes, including MYC, MDM2, CDK6, and AFR6 that are affected by silvestrol treatment making it a promising drug to treat cancer [146, 147]. In the case of breast cancer, eIF4A augments expression of an oncoprotein, mucin 1. Mucin 1 forms a complex with EGFR and activates the PI3 K-Akt-mTOR pathway [148]. Akt and p70 S6K along with activation of MEK-ERK signaling promote ubiquitination and degradation of PDCD4 [149]. This favors translation, as the MA-3 protein binding domain of PDCD4 is known to interact with eIF4A N-terminal domain and inhibit its helicase activity. Activation of the helicase activity stimulates the autoinductive pathway and increases levels of oncogenic proteins [56, 148, 150]. Further, PDCD4 inhibits eIF4A-eIF4G interaction by interacting with eIF4A as well as eIF4G. Colocalization of eIF4A and PDCD4 in the cytoplasm inhibits eIF4A helicase activity [151, 152]. eIF4A also interacts with the PAIP1 domain, containing sequence similarity to eIF4G, and promotes circularization of the mRNA, affecting protein expression involved in apoptosis [153].

As mentioned earlier, eIF4G interacts with eIF4E, eIF4A, and eIF3 to form the preinitiation complex. eIF4G consists of two binding sites for eIF4A and a single binding site for eIF4E, eIF3, PABP, Mnk1, and RNA, which interact independently of each other [154]. eIF4G interacts with PABP for effective circularization of the mRNA. Consequently, it increases the cap-binding activity of eIF4F and joining of the 60S ribosomal subunit to the 40S subunit [155–157]. Interaction of eIF4G with eIF4E enhances eIF4E-mediated cap recognition and the cap-binding activity of eIF4F complex [130]. eIF4E is associated with positive regulator HOXA9 in the nucleus. This promotes eIF4E-mediated transport of mRNAs from the nucleus to the cytoplasm. eIF4G has a higher affinity towards eIF4E in comparison to HOXA9. Therefore, HOXA9 is displaced from eIF4E in the cytoplasm to initiate translation [141].

During translation, eIF4G and eIF3 interactions bring the ternary complex onto the mRNA and stabilize the 43S preinitiation complex. These interactions are mediated by the 3c, 3d, and 3e subunits of eIF3 complex [154]. Activation of mTOR by insulin, amino acids, or growth factors influences the direct interaction between mTOR and eIF3 and increases eIF4G-eIF3 interaction. The effect on interactions is most likely mediated by phosphorylation reactions [110]. These critical interactions are extensively exploited as drug targets in cancer treatment [4].

### 4.4. eIF5

eIF5 is a 49 kDa protein in mammals and 46 kDa in* Saccharomyces cerevisiae* [158, 159]. eIF5 interacts with the 40S initiation complex to mediate hydrolysis of eIF2-bound GTP [159]. This step is critical to initiation complex formation, as the release of multiple eIFs including eIF3, eIF4E, and eIF2-GDP is necessary for the recruitment of 60S ribosomal subunit [158]. In higher eukaryotes including humans, eIF5 has a 9-residue C-terminal tail that can bind to the eIF5B-CTD subdomain [160]. This is opposed to the binding of the eIF1A C-terminus to eIF5B-CTD, which is a relatively weak interaction in both humans and yeast [160]. The interaction between eIF1A and eIF5B coordinates recruitment and release of one another in* S. cerevisiae*. However, in humans, eIF1A/eIF5B interaction facilitates subunit joining, and recruitment is coordinated separately through eIF5/eIF5B interaction [160]. eIF5 is a downstream target of casein kinase 2 (CK2), which phosphorylates eIF5 at the major sites: Ser389 and Ser390 [161]. CK2 has a significant role in cell proliferation, and both CK2 and eIF5 have critical roles in cell-cycle control and progression [161]. CK2 is necessary for the G1 and G2/M phase transitions in yeast [162]. The CK2 enzymatic activity also increases and phosphorylates eIF5, 3 hours after serum stimulation of cell-cycle arrested (G0) human embryonic kidney HEK-293 cells and normal human fetal lung fibroblasts TIG-7 cells, suggesting a role for CK2 and eIF5 in promoting cell proliferation [162]. In TIG-7 cells, eIF5 was associated with CK2. When CK2 levels were highest and when eIF5 mutants were unable to be phosphorylated by CK2, there was a decrease in growth rate, mature translation initiation complex formation, and expression of cell-cycle-regulated proteins [161]. Nuclear CK2*α* (catalytic subunit) localization is a sign of poor prognosis in prostate cancer and gastric carcinoma [163–165]. CK2s phosphorylation targets include deleted in breast cancer 1 (DBC1), eIF5, and endothelin-converting enzyme-1c (ECE-1c), which promote cancer cell invasion and progression [163–165]. Depletion of eIF5 in* S. cerevisiae* resulted in the inhibition of cell growth and a decrease in the rate of in vivo protein synthesis [159]. In yeast, eIF5 is able to mimic the effect of eIF2*α* phosphorylation, acting as a translational inhibitor and promoting translation of prooncogenic protein GCN4 (yeast equivalent of ATF4) [166, 167]. When overexpressed in* S. cerevisiae*, eIF5 increases the levels of aN eIF2/eIF5 complex, which prevents eIF2B interaction and subsequently prevents ternary complex formation [167]. eIF5-mimic protein (5MP) is a partial mimic and competition of eIF5 function. Human 5MP1 protein was found to interact with human eIF2s *β* subunit, similarly to eIF5, eIF2B*ε*, and Kra [168]. Furthermore, in vitro, eIF2*β* demonstrated mutually exclusive interactions with eIF5 and 5MP1, suggesting 5MP1 as a competitive inhibitor of eIF5 [168]. 5MP promotes expression of GADD34 (a downstream target of ATF4) in* Tribolium castaneum* [169]. Further, 5MP binds eIF2 to inhibit general translation and when overexpressed, it promotes ATF4 expression in fibrosarcoma [166, 169]. ATF4 is expressed in hypoxic- and nutrient-deprived tumor regions, with functions in development, promoting metabolic homeostasis, and cancer cell proliferation [170]. eIF5 demonstrates critical roles in cell-cycle regulation and cell proliferation with specific oncogenic protein interactions.

### 4.5. eIF5A

eIF5A is a 17 kDa protein that is activated by posttranslational hypusination and functions to mediate cell proliferation, apoptosis, and inflammatory response [171, 172]. Hypusination is unique to eIF5A and is a posttranslational enzymatic modification that involves two sequential enzymes and the substrate spermidine [173]. Deoxyhypusine synthase (DHS) catalyzes NAD-dependent cleavage and transfer of an aminobutyl moiety of spermidine to the *ε*-amino group of a conserved lysine of eIF5A [173]. The resulting intermediate residue, deoxyhypusine, is hydroxylated by deoxyhypusine hydroxylase (DOHH), yielding a hypusine residue, and an active eIF5A [173]. New treatments for chronic myeloid leukemia utilize hypusination inhibitors to deactivate eIF5A, creating a target in response to imatinib resistance towards the BCR-ABL tyrosine kinase [171]. Inhibition of eIF5A results in an antiproliferative effect on BCR-ABL positive- and negative-leukemia cell lines in vitro [171]. eIF5A is overexpressed in murine pancreatic intraepithelial neoplasia (PanIN) and in human pancreatic ductal adenocarcinoma (PDAC) [174]. Pharmacological inhibitors N(1)-guanyl-1,7,-diamineoheptane (GC7) and ciclopirox olamine (CPX) are able to inhibit DHS and DOHH, respectively, which further inhibits eIF5A hypusination and results in eIF5A genetic knockdown [174]. Genetic knockdown of eIF5A inhibited PDAC cell growth in vitro and orthotopic tumor formation in vivo, potentially through pseudopodium-enriched atypical kinase 1 (PEAK1), which is essential to PDAC tumor growth, metastasis, and gemcitabine resistance [174]. In melan-a (a murine melanocyte cell line) and Tm5 (a murine melanoma cell line derived from melan-a), GC7 was used to inhibit eIF5A [175]. More pronounced DNA fragmentation was observed in Tm5 cells and decreased viability was observed in both cell lines [175]. Additionally, treatment with GC7 was tested on melanoma growth in C57BL/6 mice and found to inhibit further tumor growth, although it did not induce volume reduction of established tumors [175]. eIF5A's isoform eIF5A2 is upregulated in various cancer types including hepatocellular carcinoma, ovarian carcinoma, and colorectal carcinoma (CRC) [176–178]. eIF5A2 overexpression in cancer cells is correlated to prognosis factors of tumor metastasis and venous infiltration [176, 177]. In ovarian carcinoma, overexpression of eIF5A2 was detected in 7% cystadenomas, 30% borderline tumors, and 53% invasive carcinomas, as opposed to normal expression in normal ovaries [177]. In CRC, eIF5A2 upregulates metastasis-associated protein 1 (MTA1) by increasing the enrichment of regulator gene c-myc on MTA1s promoter [178]. This both increases cell motility and invasion in vitro and results in tumor metastasis, inducing epithelial-mesenchymal transition and further promoting CRC aggressiveness [178]. In hepatocellular carcinoma cells in vivo, eIF5A2 suppression attenuates tumorigenic properties [176, 177]. eIF5As unique activation by hypusination creates a desirable mechanism of regulation and knockdown within various cancer lines.

### 4.6. eIF5B

eIF5B is a 175 kDa protein that promotes 60S ribosome subunit joining and pre-40S subunit proofreading and can indirectly support the tRNA-Met_*i*_ association with the ribosome in translation initiation [179–181]. In serum-starved THP1 cells, elevated eIF5B levels resulted in increased eIF5B complexes with tRNA-Met_*i*_, as well as increased phosphorylation of eIF2*α* [180]. These eIF5B complexes are formed during times of attenuated global translation, instead of promoting translation of specific stress-related mRNAs [180]. eIF5B is antagonistic of G0 and G0-like states, with eIF5B overexpression promoting maturation and cell death. eIF5B depletion is associated with increased phosphor-Cdc2, which is a marker for immaturity [180]. This suggests a critical role for eIF5B in the regulation of cell-cycle transitions [180]. eIF5B interacts with DEAD-box RNA helicase Vasa (Vas), with reduction of Vas-eIF5B interaction in* Drosophila* causing female sterility, reduced Gurken (Grk) protein levels, nearly complete loss of germ cell formation, and reduction of somatic posterior patterning [182]. eIF5B interaction with Vas is necessary for early* Drosophila* development through the progression of oogenesis and pole plasm assembly, suggesting eIF5B as a potential method to regulate Vas and subsequently Grk [182]. In poliovirus (PV) and coxsackie B virus (CBV) infection of cultured cells, eIF5B is proteolytically cleaved, suggesting that eIF5B cleavage is involved in the translation inhibition within enterovirus-infected cells [183]. Infection of enteroviruses PV and CBV begins with inhibition of host cell translation, followed by IRES-directed enterovirus protein synthesis, with eIF5B cleavage beginning at 3 hours after infection, during the attenuation of host cell protein synthesis [183, 184]. eIF5B, eIF5, and eIF5A all contain links into hallmark stages and mechanisms of cancer proliferation, and manipulation of these proteins offers a potential regulation point of gene expression regulation.

## 5. Conclusions

Mechanisms utilizing elements of protein translation, specifically in the rate-limiting step of initiation, offer potential methods to diagnose and treat cancer. Translational regulation is capable of efficiently altering specific protein levels in physiological stress conditions that are typical in cancer. Translation is mediated by eukaryotic initiation factors (eIFs), which have varying roles in regulating the rate of initiation as described in this review. The critical role of eIF2*α* phosphorylation has been classified in the integrated stress response, with the requirement of eIF4F complex for cap-binding and efficient translation through eIF4E and eIF4G. Other mechanisms include eIF3 subunit interactions with S6K1 and mTOR and eIF5A's necessary activation through posttranslational hypusination, important to the mediation of cell proliferation, apoptosis, and inflammatory response. Varying levels of eIFs in various cancer lines and stages, along with the mechanistic background, enforce the use of eIFs to regulate gene expression in cancer. During cellular stress induced by cancer, noncanonical translation utilizing uORF elements or IRES elements drives translation of specific stress response and adaptation proteins. Proteins such as ATF4 and GCN4 have critical roles in the integrated stress response and help determine whether cell proliferation ensues. The eIFs required for IRES-dependent translation are specific to the mRNA in question, with a subset of cellular IRESes not requiring eIF4G and eIF4A, while L-myc requires the full eIF4F complex and PABP. Both facilitation and inhibition of IRES-dependent translation are mediated by ITAFs. IRES-dependent translation, which specifically favors about 10 percent of cellular mRNAs under cellular stress, offers potential gene regulation targets, including various eIFs and ITAFs with critical implications for cancer treatment. The mechanisms underlying both canonical and noncanonical translation and the proteins responsible for the processes, are critical in treating the dysregulated gene expression in cancer. However, these studies are often limited in terms of what one can extract from them, since the observed phenotypical changes in nearly all levels of complexity, starting from the cellular level to the whole organism level, tend to be complicated by several other protein-protein and possibly protein-nucleic acid interactions. Future studies will need to continue building on the present data by taking mechanistic approaches in elucidating the signaling pathways, transcriptomic, translatomic, and proteomic profiles of patient samples and model systems to determine the appropriate methodology to target the function of specific regulators (e.g., proteins like initiation factors) to produce effective novel therapeutics that have intrinsically high specificity. Currently, there are several drugs and antisense oligonucleotides being tested against the initiation factors to increase mortality of cancer cells. Initiation factors, being a common element across various types of translation, hold great potential to be tested for RNA-based therapeutics as well as chemical compound-based therapeutics to treat cancer.

## Figures and Tables

**Table 1:** Differential expression of eIF3 subunits in human cancers and notable eIF3-protein interactions.

eIF3 Subunit	Expression	Cancer associations	Protein interactions
eIF3a	↑	Breast, cervix, esophagus, lung & gastric [89, 90]	ribonucleotide reductase M2 [91], RAR*α* [92], Raf-1 [93]
eIF3b	↑	Breast, bladder & prostate [90]	mTOR/S6K1 [94], ICP27 [95], DDX3 [96], NREP [97]
eIF3c	↑	Colon [98], meningioma [99] & testicular seminomas [100]	mTOR/S6K1 [94], MAPK6 [101], CDK2 [102], TARDBP [103], schwannomin [99]
eIF3d	↑	Colon [104, 105], gastric [70] & mesothelioma [70]	hTDAG51 [106], VPg [107]
eIF3e	↓	Breast & lung [89, 90]	S6K1 [94], DDX3 [96], Rpn5 [108]
eIF3f	↓	Breast, colon, melanoma & pancreas [90, 109]	mTOR [110], S6K1 [111], HnRNP K [109]
eIF3h	↑	Breast, colon, liver & prostate [89, 90]	Acetylated HIV-1 IN [112], MGMT [113]
